# Les fractures de jambe à fibula intact: traitement orthopédique ou enclouage centromédullaire? (étude comparative à propos de 60 cas)

**DOI:** 10.11604/pamj.2015.20.222.6164

**Published:** 2015-03-12

**Authors:** Merouane Abouchane, Assia Fadili, Amine Belmoubarik, Yassir EL Andaloussi, Mohammed Nechad

**Affiliations:** 1 Service de Traumatologie Orthopédie Aile IV, CHU Ibn Rochd Casablanca, Maroc

**Keywords:** Fracture, tibia, fibula, nailing, plaster, Fracture, tibia, fibula, enclouage, plâtre

## Abstract

La fracture de jambe à fibula intact (FJFI) est caractérisée par un potentiel de déplacement minime ainsi le pronostic semble bon, souvent associées à des difficultés de réduction de consolidation ou de cal vicieux. Notre but est de comparer les résultats du traitement orthopédique et de l'enclouage centromédullaire (ECM). Entre janvier 2006 et janvier 2011, on a traité 60 fractures de jambe à fibula intact. 31 patients ont bénéficié d'un ECM, dont 14 fois était statique, 16 fois dynamique et simple une fois, avec un appui compris entre le 2eme jour (pour la plupart des patients) et la sixième semaine. 29 patients ont bénéficiés d'un traitement orthopédique après réduction par plâtre cruro-pédieux avec libération du genou après 4 semaines avec appui total chez la plupart des patients. La durée de consolidation été de 4 mois en moyenne pour les patients traites par ECM et de 6 mois pour les patients traites orthopédiquement. On a noté 3 cas de pseudarthrose dans notre série d’étude soit 5%: 2 observées chez le groupe de l'ECM soit 6,4% et un cas pour le groupe du traitement orthopédique soit 3,8%. 6 cas de déplacement secondaires ont été note chez les patients traites orhtopédiquement à j15 du traitement, pour 5 patients le déplacement été jugé tolérable et pour un seul patient (trouble angulaire supérieur à 10°) ayant nécessité un ECM. 13 cas soit 54% de cal vicieux angulaire supérieur à 10 dans notre série tous traités orthopédiquement versus aucun cas de cal vicieux chez les patients du groupe du traitement par ECM. Cliniquement: tous les patients traités par ECM ont présenté une mobilité du genou et de la cheville normale. Les patients traités orthopédiquement ont tous bénéficié d'une rééducation du genou libération du genou et confection d'un plâtre type Sarmiento et celle de la cheville après son ablation définitive, ainsi aucun de nos patients n'a gardé une raideur après avoir terminé la rééducation. Le rôle de l'intégrité de la fibula dans l’évolution des fractures de jambe n'est pas négligeable, puisque sa fréquence est de 15 à 25% des fractures de jambe. Les caractères épidémiologiques, et l’évolution de chaque type de traitement (orthopédique et ECM) de ces fractures seront discuté. Il nous semble que le traitement des FJFI est l'ECM en première intention avec appui précoce et dynamisation voir fibulotomie si retard de consolidation et de réserver le traitement orthopédique pour les fractures non ou peu déplacées ou quand le traitement chirurgicale est contre indiqué.

## Introduction

L'effet du péroné intact dans l’évolution des fractures du tibia était et reste toujours un sujet de débat, quelques auteurs croient que son intégrité contribue à la stabilité de la fracture ce qui permet de meilleurs conditions de consolidation [1, 2]. Au contraire, Jackson et Macnab [3] et Charnley [4] trouvent que la consolidation est retardée lorsque le péroné est continu allant engendrer un déplacement en valgus et diminuer les contraintes compressives axiales. Pour Sarmiento [5] expert en traitement orthopédique des fractures de jambe retrouve que l'intégrité de la fibula retarde cette consolidation. D'autres auteurs rapportent d'excellents résultats après enclouage centromédullaire (ECM) dans le traitement des fractures de jambe [6–17] notamment Bonnevialle [18] qui à publié récemment à propos du sujet. L'objectif de cette étude prospective est de comparer les résultats cliniques et radiologiques de l'ECM et du traitement orthopédique dans le traitement des fractures de jambe à péroné intact.

## Méthodes

A travers une étude prospective nous avons suivis 60 patients de janvier 2006 à janvier 2011, traités au sein du service de traumatologie orthopédie Aile 4 du CHU Ibn Rochd à la ville de Casablanca. L’âge moyen de nos patients été de 25 ans avec des extrêmes de 19 et 59 ans, à prédominance masculine (5 hommes / 1 femme), le cote atteint été gauche chez 31 patients (52%) contre 29 patients atteints du coté droit (48%). Les étiologies du traumatisme étaient dominées par les AVP dans 57% des cas suivies par les accidents de sport avec 22% des cas (football + + +). 5 de nos patients ont présenté des lésions associées: 2 fractures de fémur homolatéral réalisant 2 genoux flottants, 2 fractures de la jambe controlatérale et une fracture de l'avant bras du coté controlatérale. Les fractures ouvertes nécessitant une fixation externe ont été exclus de notre étude, du coup on a inclus 5 fractures avec ouverture cutanée de type I. Les radiographies de la jambe de face et de profil prenant le genou et la cheville ont été demandées pour juger du siège, le type de fracture, le déplacement et pour classer les fractures. La classification utilisée était celle de l'AO retrouvant: 45 fractures (soit 74%) de type A, 10 fractures (soit 17%) de type B et 5 fractures (soit 9%) de type C. La localisation de la fracture été jugée par rapport au 3 tiers de la diaphyse tibial: proximal, moyen et distal, ainsi 49 fractures ont siégé au niveau du 1/3 moyen, 8 fractures au niveau du 1/3 distal et 3 fractures au niveau du 1/3 proximal. Le déplacement des fractures été jugé sur la radiographie de face et de profil, cette analyse a retrouvé: 14 fractures non déplacées sur la face et sur le profil, sur la face (22 fractures en translation, 8 en valgus, 8 en varus et 22 sans déplacement sur la face), sur le profil (8 déplacement en translation, 2 en recurvatum, 6 en flessum, et 44 non déplacées sur le profil). La consolidation été jugé cliniquement par absence de douleur et mobilité du foyer fracturaire et radiologiquement par la présence d'un cal uniforme sur la face et le profil. Une pseudarthrose été considérée si on a une absence de consolidation après le 6 éme mois du traitement. La décision de consolidation été déterminé par un professeur senior. Deux méthodes thérapeutiques ont été adoptées: le traitement orthopédique avec réduction à foyer fermé, et ECM. Le traitement par ECM été indiqué chez 31 patients dont 30 de première intention et 1 après échec du traitement orthopédique (déplacement secondaire à 15 j après la mise en place du plâtre).

**Figure 1:**
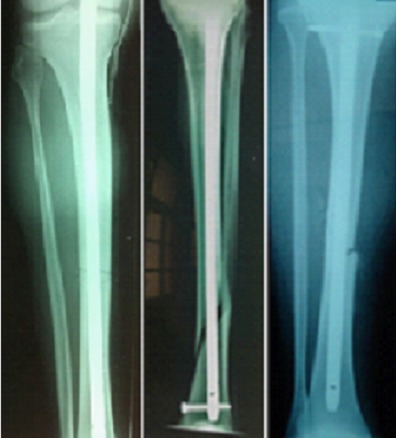
(A): ECM simple B1 et B2: ECM dynamique

**Figure 2:**
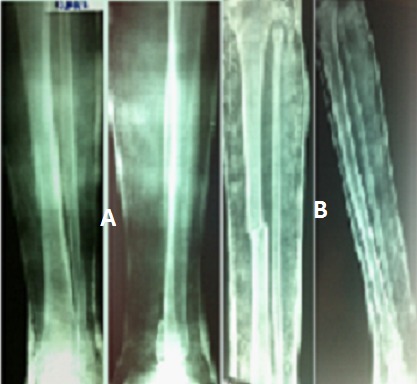
Traitement orthopédique: (A): fracture type A strictement non déplacée; (B): fracture de type A déplacée en translation avec contact sup. à 50% sur la face

L'ECM été réalisé entre le 3ème et le 7ème jour (en fonction de l’état cutanée et la disponibilité du matériel) après l'hospitalisation et ceci après réduction et immobilisation du membre par attelle cruro pédieuse. Tous les clous on été introduit après alésage, avec un diamètre de 10 ou 11 mm. Le type de verrouillage été détermine par la localisation de la fracture et sa stabilité (Figure 1). Les fractures jugées instables étaient systématiquement verrouillées statiquement pour prévenir tout déplacement secondaire. Ainsi 14 de nos fractures on été verrouillées statiquement (Tableau 1). Le suivi été assuré 15 jours après l'opération puis chaque mois. L'appui été déterminé par la stabilité de la fracture et du montage, le degré de la comminution, les lésions associées et le confort du patient. Ainsi il été autorisé d'appuyer depuis le lendemain jusqu’à la sixième semaine. Les patients traités orthopédiquement, ont bénéficié d'une réduction jugée satisfaisante (contact plus de 50% des deux corticales sur la radiographie de face et de profil, une angulation ne déplaçant pas 10° dans toutes les directions et sans trouble rotatoire) ou présentant une fracture non déplacée (Figure 2A et B), avec mise en place d'un plâtre cruro pédieux et surélévation du membre avec hospitalisation pour surveillance. À savoir qu'aucune fibulotomie n'a été pratiqué pour avoir une réduction satisfaisante. Le plâtre été mis en place pendant 12 semaines avec libération du genou à la 4ème semaine. L'appui été autorisé à partir de la 4ème semaine jusqu’à la 8éme semaine en fonction des caractéristiques de la fracture et du patient. Le suivi des patients traités par ECM: 15 jours après enclouage, puis tous les mois jusqu’à consolidation; pour les patients traites orthopédiquement: ils ont été vu tous les 15 jours pendant 6 semaines puis tous les mois jusqu’à consolidation. Le recul été de deux ans en moyenne, nos critères d’évaluation clinique étaient: la mobilité du genou et de la cheville et la douleur lors de l'appui.

**Table 1:** Type de montage dans notre série

Type AO	Montage statique	Montage dynamique	Montage simple
Type A	7	14	1
Type B	5	2	
Type C	2		
Total	14	16	1

## Résultats

**Figure 3:**
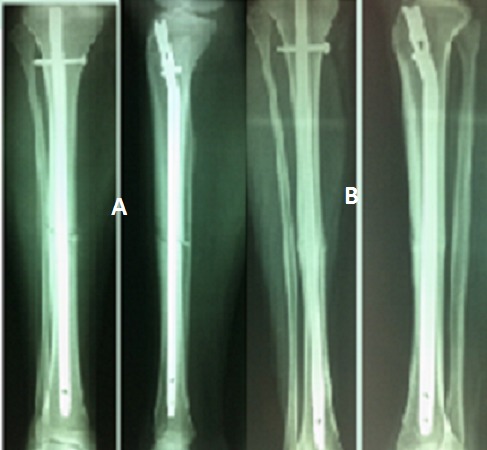
(A): contrôle à 1 mois; (B): contrôle à 4 mois

**Figure 4:**
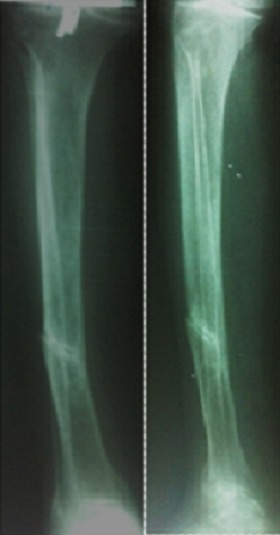
Contrôle de traitement orthopédique à 6 mois

**Figure 5:**
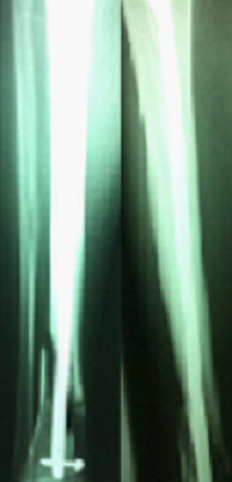
Pseudarthrose aseptique à 1 an après ECM

**Figure 6:**
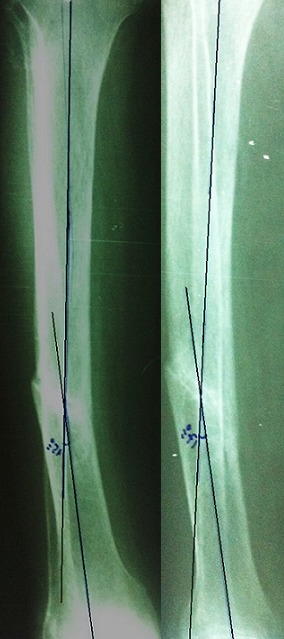
Cal vicieux après traitement orthopédique: varus:12°; recurvatum:17

La durée de consolidation été de 4 mois (16 semaines) en moyenne pour les patients traites par ECM (Figure 3) et de 6 mois (24 semaines) en moyenne pour les patients traites orthopédiquement (Figure 4). Aucun syndrome de loge n'a été noté dans notre étude. On a noté 3 cas de pseudarthrose dans notre série d’étude soit 5%: 2 observées chez le groupe de l'ECM soit 6,4% et un cas pour le groupe du traitement orthopédique soit 3,8%. Pour l'ECM un patient a présenté une pseudarthrose septique alors que pour l'autre c’été aseptique (Figure 5) identique à celui du groupe du traitement orthopédique. 6 cas de déplacement secondaires ont été note chez les patients traites orhtopédiquement à j15 du traitement, pour 5 patients le déplacement été jugé tolérable et pour un seul patient (trouble angulaire supérieur à 10°) ayant nécessité un ECM. 13 cas soit 54% de cal vicieux angulaire supérieur à 10° (10 en varus recurvatum et 3 en valgus) dans notre série tous traités orthopédiquement (Figure 6) versus aucun cas de cal vicieux chez les patients du groupe du traitement par ECM. Aucun trouble rotatoire n'a été note. Cliniquement: tous les patients traités par ECM ont présenté une mobilité du genou et de la cheville normale, dont 10 présentent des douleurs jugées tolérables au niveau du point d'entré du genou. Les patients traités orthopédiquement ont tous bénéficié d'une rééducation du genou libération du genou et confection d'un plâtre type Sarmiento et celle de la cheville après son ablation définitive, ainsi aucun de nos patients n'a gardé une raideur après avoir terminé la rééducation. L'interrogatoire de quelques patients traités orthopédiquement a révélé qu'ils été gênés au court du traitement et ils ne sont pas prêts à refaire le même traitement au contraire de ceux traités par ECM satisfaits du traitement.

## Discussion

Le rôle de l'intégrité de la fibula dans l’évolution des fractures de jambe n'est pas négligeable, puisque sa fréquence est de 15 à 25% des fractures de jambe selon la littérature, 9% dans la série de Bejui [19] et 24% dans la série de Sarmiento [5]. (Voir Tableau 2) Sur le plan épidémiologique ce type de fracture est l'apanage des sujets jeunes, surtout la 3eme décennie [18]. Les étiologies sont dominées par les accidents de sport; le football vient au premier rang avec un choc qui est souvent direct [14] et de faible énergie [18], dans notre série on a retrouvé plus d accidents de la voie publique peut être le contexte de notre pays où les accidents de la voie publique (1er au rang des pays arabes et 6 éme mondial) comparable à celle de Bonvaille [18]. L'ouverture cutanée est en général est un type I selon Hooper [14] 19% de fractures ouvertes toutes de type I, Bonvaille [18] rapporte 10 fractures ouvertes dans une série de 38 dont 7 de type I) La fracture est dans presque 50% des cas transversale simple médiodiaphysaire [14] et Bonnevialle [18] et pourvues d'un potentiel de déplacement très minime (chez Hooper [14] 55% des fractures de sa série étaient non déplacés et 19% chez Bonnevialle [18]). Quelle attitude thérapeutique devant ce genre de fracture? Ca reste toujours un sujet de discussion; plusieurs auteurs recommandent le traitement orthopédique pour ce genre de fracture, en s'appuyant sur plusieurs arguments: le risque infectieux de l'ostéosynthèse, une consolidation plus rapide que lorsque le péroné est fracturé (16 semaines pour Sarmieto [5]) et le taux de pseudarthrose qui est moindre par rapport au traitement chirurgicale (1,8% pour Hooper [14]).

**Table 2:** Fréquence des fractures de jambe à péroné intact dans les séries récentes de fractures de jambe

Auteurs	Nombre de fractures	Pourcentage
BEJUI (19)	100	9
SARMIENTO (5)	780	24

**Figure 7:**
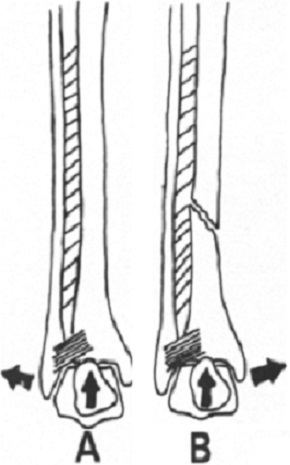
Rôle de la dorsiflexion de la cheville dans le déplacement de la fracture du tibia

Actuellement on se penche de plus en plus vers le traitement chirurgicale par ECM, en comptant les aléas du traitements orthopédique [18] à savoir la lenteur de consolidation et le déplacement secondaire rencontré dans 40 à 50% des cas et ceci même si la fracture est non déplacée au début du traitement, Odoyer [20] explique ce déplacement secondaire qui se fait en général en varus, par la dorsiflexion de la cheville au cours du traitement orthopédique (Figure 7) et pour palier cet inconvénient un plâtre en équin est indiqué mais la marche sera gênée voir impossible avec risque de garder un équin après ablation du plâtre. Les résultats cliniques sont meilleurs que le traitement orthopédique avec une bonne mobilité du genou et de la cheville [18], sans cal vicieux. Par ailleurs les auteurs rapporte un retard de consolidation plus long que le traitement orthopédique [18] mais dans notre série ca n'a pas été démontré (peut être parce qu'on a retardé l'appui à 4 semaines au minimum), aussi l'ECM est pourvu d'un taux de pseudarthrose plus important que le traitement orthopédique, qui été de 5.2% pour Bonnvaille [18], ceci peut être du au risque infectieux de l'enclouage ainsi que le type de montage, mais dans toute suspicion de retard de consolidation il faut pas hésiter à dynamiser voir même fibulotomiser avec appui précoce [18]. Au terme de cette étude il nous semble que le traitement des FJFI est l'ECM en première intention avec appui précoce et dynamisation voir fibulotomie si retard de consolidation et de réserver le traitement orthopédique pour les fractures non ou peut déplacées ou quand le traitement chirurgicale est contre indiqué.

## Conclusion

Les fractures isolées de la diaphyse tibiale sont classiquement traitées orthopédiquement ayant la fausse réputation de bénignité, tandis que le traitement chirurgicale est réservé aux situations d’échec du traitement orthopédique soit par difficulté d'avoir ou de maintenir une réduction à foyer fermé. Ces dernières années, d'excellents résultats ont été rapportés par un certain nombre d'auteurs après enclouage centromédullaire, permettant un appui précoce, un délai de consolidation moindre et une qualité de vie meilleure.

## References

[1] Nicoll EA (1964). Fractures of the tibia1 shah. J Bone Joint Surg..

[2] Hoaglund FT, States JD (1967). Factors influencing the rate of healing in tibia1 shaft fractures. Surg Gynecol Obstet..

[3] Jackson RW, Macnab I (1959). Fractures of the shaft of the tibia; a clinical and experimental study. Am J Surg..

[4] Charnley J (1961). The Closed Treatment of Common Fractures.

[5] Sarmiento A (1967). A functional below-the-knee cast for tibia1 fractures. J Bone Joint Surg Am..

[6] Sarmiento A (1970). A functional below-the-kneebrace for tibia fractures. J Bone Joint Surg Am..

[7] Sarmiento A (1972). Functional bracing of tibia1 and femoral shaft fractures. Clin Orthop Relat Res..

[8] Alho A, Benterud JG, Hogevold HE, Ekeland A, Stromsoe K (1992). Comparison of functional bracing and locked intramedullary nailing in the treatment of displaced tibial shaft fractures. Clin Orthop Relat Res..

[9] Bone LB, Johnson KD (1986). Treatment of tibial fractures by reaming and intramedullary nailing. J Bone and Joint Surg..

[10] Bostman OM (1986). Spiral fractures of the shaft of the tibia Initial displacement and stability of reduction. J Bone Joint Surg Br..

[11] Digby JM, Holloway GM, Webb JK (1983). A study of function after tibial cast bracing. Injury..

[12] Collins DN, Pearce CE, McAndrew MP (1990). Successful use of reaming and intramedullary nailing of the tibia. J Orthop Trauma..

[13] Ekeland A, Thoresen BO, Alho A, Stromsoe K, Folleras G, Haukebo A (1988). Interlocking intramedullary nailing in the treatment of tibial fractures: A report of 45 cases. Clin Orthop Relat Res..

[14] Hooper GJ, Keddell RG, Penny ID (1991). Conservative management or closed nailing for tibial shaft fractures A randomized prospective trial. J Bone Joint Surg Br..

[15] Olerud S, Karlstrom G (1986). The spectrum of intramedullary nailing of the tibia. Clin Orthop Relat Res..

[16] Puno RM, Vaughan JJ, Stetten ML, Johnson JR (1991). Long-term effects of tibial angular malunion on the knee and ankle joints. J Orthop Trauma..

[17] Trafton PG (1988). Closed unstable fractures of the tibia. Clin Orthop Relat Res..

[18] Bonnevialle P, Bellumore Y, Foucras L, Hézard L, Mansat M (2000). [Tibial fracture with intact fibula treated by reamed nailing]. Rev Chir Orthop Reparatrice Appar Mot..

[19] Bejui J, Carret JP, Fischer LP, Berger E, Bertrand HG, Lille R, Chadenson O, Benoit Y (1982). Etude critique de l'enclouage du tibia avec alésage et à foyer fermé: A propos d'une série continue de 100 cas. Rev Chir Orthop Reparatrice Appar Mot..

[20] O'Dwyer KJ, Devriese L, Feys H, Vercruysse L, Jameson-Evans DC (1992). The intact fibula. Injury..

